# PS-08 - UKHSA FIELD SERVICES RAPID INVESTIGATION TEAM: ACTIVITY AND EARLY IMPACT

**DOI:** 10.1093/pubmed/fdag046.057

**Published:** 2026-07-09

**Authors:** Mrs Oluwakemi Olufon, Mr Neil Bray, Ms Carmellie Inzoungou-Massanga, Ms Florence Halford, Ms Eve Matthews, Ms Anita Shah, Mr Motolani Awokoya, Mr Saravana Kumar Kothandan, Ms Deepti Kumar

**Affiliations:** UK Health Security Agency; UK Health Security Agency; UK Health Security Agency; UK Health Security Agency; UK Health Security Agency; UK Health Security Agency; UK Health Security Agency; UK Health Security Agency; UK Health Security Agency

## Abstract

**Background:**

The Field Services Rapid Investigation Team (RIT), established in 2022, is the UK Health Security Agency’s (UKHSA’s) national, multidisciplinary, deployable rapid response service. RIT adds to UKHSA’s response capability, working alongside regional Health Protection Teams and the global PHRST to respond to complex and enhanced incidents. RIT uniquely supports domestic incidents by bringing together field epidemiology, biological sampling and operational expertise.

**Objective:**

We aim to share RITs capabilities, activation model, challenges, lessons learnt and achievements to date. We aim to highlight the significant contributions the RIT has made to national incident response to date.

**Methods:**

We reviewed and summarised data on RITs biological sampling and epidemiological input for national incidents, enhanced surveillance programmes and research activities. We also reviewed RITs skill capabilities and future scope.

**Results:**

The team consists of a Consultant Epidemiologist, Health Protection Practitioners (HPPs), Epi-Scientists, and business support staff. Clinical staff and Epi-Scientists provide an integrated capability of data collection, rapid data analysis, report production and field biological sampling. Data collection tools are designed by the Epi-Scientists and conducted by the HPPs through online or face to face interviews. HPPs are responsible for collecting biological samples in response to an incident.

Since September 2022, the team has responded to over 60 complex and enhanced incidents for UKHSA, and has deployed to 25 sites for sample collection. These incidents included numerous high-profile national investigations such as mpox, rabies, measles and most recently meningococcal B. RIT has operationalised an asymptomatic zoonotic influenza study, and are currently operationalising academic studies for Group A Streptococcal outbreaks in schools and nurseries as well a carriage study for meningococcal infection in university students.

**Conclusions:**

RIT is a high impact team which provides a flexible, evidence-driven capability that improves national readiness for “all-hazards” threats, supports rapid decision-making and strengthens UKHSA’s preparedness capabilities.

